# Terminology and methods used to differentiate injury intent of hospital burn patients in South Asia: a systematic scoping review protocol

**DOI:** 10.1186/s13643-023-02317-y

**Published:** 2023-08-31

**Authors:** Emily Bebbington, Parvathy Ramesh, Mohan Kakola, Rebecca McPhillips, Fatima Bibi, Atiya Hanif, Nia Morris, Murad Khan, Rob Poole, Catherine Robinson

**Affiliations:** 1 Centre for Mental Health and Society, School of Medical and Health Sciences, Bangor University, Bangor, LL13 7YP UK; 2 Division of Psychology and Mental Health, School of Health Sciences, University of Manchester, Manchester, UK; 3 Department of Plastic Surgery and Burns, Mysore Medical College and Research Institute, Mysuru, India; 4 Social Care and Society, School of Health Sciences, University of Manchester, Manchester, UK; 5 Turning Point, 5 Greaves Street, Oldham, UK; 6 Lancashire County Council, Burnley, UK; 7 John Spalding Library, Wrexham Maelor Hospital, Wrexham, UK; 8 Brain and Mind Institute, Aga Khan University, Karachi, Pakistan

**Keywords:** Burns, Surveillance, Injury, Intent, Systematic scoping review

## Abstract

**Background:**

The greatest proportion of burn injuries globally occur in South Asia, where there are also high numbers of intentional burns. Burn injury prevention efforts are hampered by poor surveillance data on injury intent. There is a plethora of local routinely collected data in the research literature from South Asia that could be used for epidemiological purposes, but it is not known whether the definitions and methods of differentiation of injury intent are sufficiently homogenous to allow valid study comparisons.

**Methods:**

We will conduct a systematic scoping review to understand terminology and methods used to differentiate injury intent of hospital burn patients in South Asia. The objectives of the study are to: determine the breadth of terminology and common terms used for burn injury intent; to determine if definitions are comparable across studies where the same term is used; and to appraise the rigour of methods used to differentiate burn injury intent and suitability for comparison across studies. The databases Embase, MEDLINE, CINAHL, PsycINFO, and PakMediNet will be searched. Screening and data extraction will be completed independently by two reviewers. To be included, the article must be as follows: peer reviewed, primary research, study cutaneous burns, based on hospital patients from a country in South Asia, and use intent terminology or discuss a method of differentiation of injury intent. Results will be restricted to English language studies. No date restrictions will be applied. A plain language summary and terminology section are included for non-specialist readers.

**Discussion:**

Results will be used to inform stakeholder work to develop standardised terminology and methods for burn injury intent in South Asia. They will be published open access in peer-reviewed journals wherever possible.

**Systematic review registration:**

This review has been registered with the Open Science Framework (https://doi.org/10.17605/OSF.IO/DCYNQ).

**Supplementary Information:**

The online version contains supplementary material available at 10.1186/s13643-023-02317-y.

## Background

Burn injuries are a major source of morbidity globally. Estimation of the number of burn injuries is complicated by the numerous mechanisms by which a burn can be sustained (e.g. hot objects, chemicals, friction). The Global Burden of Disease study estimates that in 2019 there were 16.3 million burn injuries from all causes [1]. 3.7 million are thought to have occurred in South Asia, the vast majority of these in India, but this may be an underestimate [1, 2]. Similar under-reporting may affect other countries where there is no national injury surveillance system. Burn morbidity surveillance statistics are compiled from hospital-based data [3]. A cornerstone of burn injury surveillance is hospital-based registers of burn presentations, which collect data on burn injuries in a standardised manner across institutions. There are no national registers in South Asia, though one has been proposed for India and there has been a successful pilot in Pakistan and Bangladesh [4, 5]. The World Health Organization Global Burn Register allows any hospital that admits burn patients to submit data, but these data are submitted voluntarily by individual hospitals rather than national sources, meaning data are unlikely to be representative of the entire population of a country [6].

Intent is the first level of classification of an injury, deemed to be the most useful for identifying intervention opportunities and thus should be part of any minimum dataset [7, 8]. The International Classification of Diseases (ICD) external causes chapter defines this concept as ‘whether or not they [injuries] were deliberately inflicted and by whom’ (e.g. unintentional, intentional self-harm, assault, undetermined intent, maltreatment) [7]. Information about burn injury intent that is used for surveillance purposes globally is typically collected from hospital records [9]. This information is documented by the healthcare professional caring for the patient. Collection of this information is influenced by personal, social, religious, and legal sensitivities, creating a bias for burn injuries to be misclassified in routinely collected data [10]. Patients may be reluctant to disclose the true cause of an injury in cases of assault [11–13]. Factors that contribute to misreporting include domestic coercive control, criminal investigation, personal safety, ‘honour’, and financial dependency [11–15]. Self-inflicted injuries may not be reported due to stigma or fear of criminal investigation, particularly in countries where suicide has not been decriminalised [15–18]. There are variable requirements internationally for hospitals to report injuries due to self-harm or assault to the police [19]. These factors also affect the accuracy of clinicians’ documentation [20].

Local studies suggest South Asia has the highest proportion of intentional burns in the world [21]. Prevention of burn injuries in South Asia is hampered by unreliable population-level estimates due to poor surveillance systems, lack of data disaggregation by injury intent, unreliable classification of intent where data are collected, and incomplete sharing of data [22, 23]. There is a wealth of routinely collected data and primary research data on injury intent in the peer-reviewed literature from South Asia that could potentially be utilised for surveillance purposes. However, little work has been done to understand how these data have been collected and if the methods are comparable across studies. Differences in definitions or method of differentiation of intent may act as sources of bias if attempting to compare data across studies. We will conduct a systematic scoping review to understand terminology and methods used to differentiate injury intent of hospital burn patients in South Asia.

### Study objectives

1. Determine the breadth of terminology and most commonly used terms for burn injury intent, including the stem term and classifiers.
2. Determine if definitions are comparable across studies where the same term is used.
3. Appraise the rigour of methods used to differentiate burn injury intent and suitability for comparison across studies.

A preliminary search of MEDLINE and PROSPERO was conducted, and no systematic reviews or scoping reviews on the topic were identified.

## Methods

### Protocol and registration

This protocol has been written using Preferred Reporting Items for Systematic Review and Meta-Analysis Protocols (PRISMA-P) (see Additional file 1) and Preferred Reporting Items for Systematic Reviews and Meta-Analyses extension for Scoping Reviews (PRISMA-ScR) [24, 25]. PRISMA does not have a protocol guideline for scoping reviews. This review has been registered with the Open Science Framework (https://doi.org/10.17605/OSF.IO/DCYNQ).

### Eligibility criteria

#### Population

Articles that present data on patients with a cutaneous burn injury will be included. A burn is defined as “an injury to the tissues caused by a pathological flux of energy which causes cellular destruction and irreversible denaturation of proteins and is primarily caused by thermal or other acute trauma [e.g. chemical, electricity, friction]” [26]. Studies that focus exclusively on ocular burns (e.g. chemical burn to the eye) or internal burns (e.g. oesophageal burn from ingestion of corrosive substances) will be excluded because such burns are unlikely to be looked after in burn specialist services. Burns sustained during combat (e.g. blast injury, military casualties) will be excluded because the concept of intent during armed conflict is distinct from intent during peacetime. Articles not related to burn injuries (e.g. professional burnout, heartburn) will be excluded.

#### Concept

**Fig. 1:**
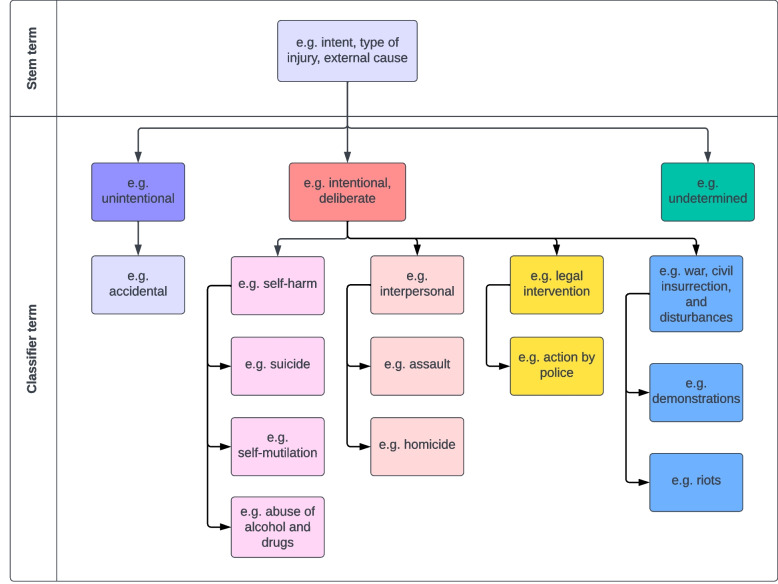
Example of how terms used in the International Classification of Diseases 11th Revision external causes of morbidity or mortality chapter could be split into stem and classifier terms [7]

Studies will be included that use a term referring to intent (e.g. intent, motive) or its classification (e.g. unintentional, intentional, accidental, homicidal, suicidal, undetermined). These will be referred to as ‘stem’ and ‘classifier’ terms, where stem is overarching term for the concept, and classifier is a type of response option (Fig. 1). Articles using an ambiguous term (e.g. aetiology, cause, circumstances of the injury) will be included at the title and abstract screening phase and undergo full-text review. Reviewers are not restricted to these terms and can infer meaning from the rest of the abstract or article. Studies that do not include information on burn injury intent will be excluded.

#### Context I: hospital

Studies will be included that have taken place in hospital. This will be decided at the title and abstract screening stage, where the article should include the term ‘hospital’ or equivalent (e.g. secondary care, tertiary care). If the study does not explicitly state data was collected from a hospital but it is inferred, then it will be included at the title and abstract screening phase for full-text review. Articles will be excluded where data has not been collected from hospital patients (e.g. autopsy studies, coroner’s studies, medicolegal death studies).

#### Context II: South Asia

Studies will be included that have taken place in South Asia. We will use the World Bank definition for South Asia, which includes the countries of Afghanistan, Bangladesh, Bhutan, India, Sri Lanka, Maldives, Nepal, and Pakistan [27, 28].

#### Study design

Only peer-reviewed publications based on primary research data will be included (e.g. quantitative studies, qualitative studies, case series, and case reports). Articles that do not present original data (e.g. review articles, opinion pieces, and personal practice) and grey literature (e.g. unpublished works, published conference abstracts) will be excluded due to the large volume of relevant peer-reviewed publications with original data identified in the preliminary search.

#### Report characteristics

There will be no restriction on the year of publication. Results will be restricted to human studies. Research conducted by South Asian researchers is almost always published in English [29]. Therefore, search results will be restricted to English language papers.

### Information sources

Searches will be conducted using the major medical and social science databases Embase, MEDLINE, CINAHL, and PsycINFO. PakMediNet will be used to search peer-reviewed Pakistani medical journals that may not be indexed in the aforementioned databases. No date restrictions will be applied.

### Search strategy

An initial search strategy was developed for key concepts from the review question (burns, hospital, South Asia) in MEDLINE. MESH headings and keywords were identified for each concept. Each term was looked up in an online dictionary to identify similarly spelt words. This dictated which terms were truncated and excluded. An initial limited search of MEDLINE was undertaken using the search strategy. The results were cross-referenced against previously identified articles of interest. Search terms were modified to include index terms of articles that had not been identified in the preliminary search. The preliminary search was repeated using the key concepts ‘burns’ and ‘South Asia’ but excluding ‘hospital’. Results were screened for any articles of interest that were missed by inclusion of the concept ‘hospital’. The text words contained in the titles and abstracts of relevant articles, and the index terms used to describe the articles, were used to further refine the full search strategy for MEDLINE (see Additional file 2). The search strategy, including all identified keywords and index terms, will be adapted for each included database.

### Study records

Searches of Embase, MEDLINE, and PsycINFO will be completed using the Ovid platform. Searches of CINAHL will be completed using the EBSCO platform. Searches of PakMediNet will be completed using the database website. All searches will be saved in a user account. A separate record of the search results will be kept in a spreadsheet file including date of search, search terms, and number of retrieved articles. Search results will be exported into reference management software EndNote X9. Duplicate articles will be removed using the method by Bramer et al. [30]. Remaining references will be uploaded into systematic review software Covidence (Covidence systematic review software, Veritas Health Innovation, Melbourne, Australia. Available at www.covidence.org). Title and abstract screening, and full-text review, will be completed by two researchers using Covidence (E. B., P. R.). Any disputes will be resolved by a third researcher (R. M.). Authors undertaking study screening will undergo training to ensure the study objectives are understood and a standardised process is followed (see Additional file 3). The reason for full-text article exclusion will be recorded in Covidence based upon a hierarchy of predefined criteria:

- Duplicate
- Not in English
- Non-human study
- Not a peer-reviewed publication
- No original data presented (e.g. not a primary research study)
- Cutaneous burns not studied (e.g. focus of article are ocular/oesophageal burns)
- Study not from a country in South Asia
- Study not based on hospital patients (e.g. autopsy study)
- Burns sustained during combat
- Intent terminology or method of differentiation not discussed

Results from each of these steps will be downloaded as a spreadsheet file from Covidence. Full-text copies of all included articles will be uploaded into Covidence for data extraction. Extracted data will be entered into a template on specially designed template in Covidence. Two reviewers (E. B., P. R.) will extract data from the full-text articles individually. The results will then be compared and discrepancies resolved by discussion. Reviewers will meet regularly to discuss any issues that may have arisen. Any deviation from the review protocol will be recorded and reported in the results manuscript.

### Data items

Data items will include the following: study name, journal name, date of publication, first author name, institution(s) where the study took place, dates of study, study aim, number of participants, age range of participants, stem term(s) used for burn injury intent (e.g. intent, aetiology, motive, cause), definition of stem term(s) used for burn injury intent, classifier term(s) used for burn injury intent (e.g. unintentional, intentional, accidental, homicidal, suicidal, undetermined), definition of classifier term(s) used for burn injury intent, method used to differentiate burn injury intent, role of person completing assessment (e.g. healthcare professional, researcher), and timing/location of burn injury intent assessment in the patient’s hospital journey (e.g. emergency department triage, arrival at burns ward, upon discharge from burns ward). Where data items are missing or not applicable to a study, the research will assign a code (e.g. NA — not applicable, INIS — information not in study). This is to ensure missing data is not attributed to the data entrant. A spreadsheet file with all extracted data will be downloaded from Covidence for data analysis and synthesis.

### Risk of bias in individual studies

The risk of bias overall in individual studies will not be assessed beyond assessing rigour of the method used differentiate injury intent.

### Data synthesis based on study objectives

#### Determine the breadth of terminology and most commonly used terms for burn injury intent, including the stem term and classifiers

A list of stem terms with the number of papers using each term will be compiled using spreadsheet software. A full list of terms will be presented as supplementary data. The top five terms will be presented in tabular format in the results manuscript. The same process and presentation of results will be completed for classifier terms.

#### Determine if definitions are comparable across studies where the same term is used

Definitions for each stem term will be tabulated with the corresponding study. The number of times a term is not defined will also be presented. Full results will be presented as supplementary data. Examples of comparable and incomparable definitions for terms will be presented in the results manuscript. The same process and presentation of results will be completed for classifier terms.

#### Appraise the rigour of methods used to differentiate burn injury intent and suitability for comparison across studies

The method of differentiation of burn injury intent by each study will be compiled in tabular format. Studies using the same methods will be presented together. A list will be compiled of studies that do not include details about the method of differentiation of intent. A table with summary data will be included in the results manuscript. Full results will be presented as supplementary data.

The rigour of the method used to determine burn injury intent will be appraised by a modification of the method described by Maguire et al. [31]. This method was developed for ranking quality of evidence for identification of paediatric burns caused by abuse. Consequently, there is no ranking system for burns due to self-harm. For cases of assault (or equivalent term for interpersonal violence), the method of differentiation will be ascribed a ranking between 1 and 5 (1 — assault confirmed at case conference, or court proceeding, or admitted by perpetrator; 2 — assault confirmed by stated criteria including multidisciplinary assessment; 3 — diagnosis of assault defined by stated criteria; 4 — assault stated as occurring but no supporting detail given as to how it was determined; 5 — abuse stated as “suspected” with not details given on whether it was confirmed or not). For accidental burns (or equivalent term for unintentional injury), the method of differentiation will be ascribed a ranking A–C (A — scene of incident recreated or forensic police investigation of scene or criminal investigation ruled out assault as a cause; B — efforts specifically made to exclude assault as a cause for burn through multidisciplinary investigation; C — no discussion about how burn was deemed to be accidental). Summary statistics for each category will be presented in tabular format. There is no plan to complete assessment of meta-biases or the strength of the body of evidence.

### Patient and public involvement

This protocol has been reviewed by two people who were not researchers but do have volunteering and practice experience in the fields of child protection, adult safeguarding, criminal justice, and substance misuse. Both will be involved in the analysis and write up of the results manuscript to ensure the paper is accessible and service user focussed. We have included a summary and terminology section for non-specialist readers to increase the accessibility and usability of this article.

### Plain language summary

This article describes how we plan to review some research. We wish to look at articles that include patients from South Asia with a *burn injury*. We are interested in the words that are used to describe the *intent* of the injury, how those words are defined, and how intent was determined. Injury intent often includes if the injury was caused on purpose and who did it. For example, the injury could be classed as an accident, self-harm, or assault. We will review existing literature using a method known as a *systematic scoping review*. This is a standardised way to review lots of research articles. We have described how we will do the review so that it can also be understood by others and repeated. It is important to do this review because it is believed that most burns due to self-harm and assault happen in South Asia. There is not much data collected about this at a national level (*surveillance*). This data is needed to develop ways to prevent burns occurring in the first place. Lots of hospitals in South Asia publish their own data on burn injuries. If the data is collected in similar ways, it might be able to be used for surveillance. This is why we want to understand how the words for intent are used, defined, and measured in the articles.

## Discussion

There are a number of strengths in the methods of this planned systematic scoping review. PRISMA protocol and scoping review guidelines have been followed for the preparation of this protocol. An information specialist is part of the author team and has helped to devise a robust search strategy. Two members of the public have co-produced this research to ensure it is suitably service user focussed, as well as helping to create a glossary and summary for non-specialist readers to ensure the article is accessible. We plan to appraise the rigour of method to determine burn injury intent. There is no accepted method to do so in adults, so a method devised for paediatric populations has been adapted for this review. A database with good coverage of social science papers (e.g. Scopus) has not been used. This is a potential limitation of the review.

This review process is expected to generate peer-reviewed publications on terminology and operational criteria for classifying burn injury intent. This includes recommendations for standard terminology, methods of differentiation of burn injury intent, recommendations on minimising misclassification, and an assessment of whether the literature is sufficiently methodologically consistent to allow valid inter-study comparisons. Publications will be open access wherever possible to ensure results are accessible to clinicians and stakeholders in South Asia, as well as the public. Results will provide the basis for stakeholder engagement in international consensus work on standardised terminology and methods for burn injury intent in South Asia.

### Terminology

Key terms used in this protocol have been defined for readers who are not specialists in the field (Table 1).

**Table 1:** Description of key terms used in this article that may be useful for non-specialist readers

Term	Description
Burn injury	Burns are a type of injury to the skin (cutaneous burn) or other type of body tissue (e.g. eye — ocular burn). They may be caused by heat (thermal burn), chemicals, radiation, electricity, or friction [32].
Surveillance	Surveillance is a key aspect of public health. It is the practice of collection, analysis, and reporting of data on injuries and diseases. These data provide timely information used to set government priorities and inform methods to stop injuries and diseases [33].
Injury intent	This review aims to understand the breadth of definitions of the concept of injury intent in South Asia. The International Classification of Diseases (ICD) defines this concept as ‘whether or not they [injuries] were deliberately inflicted and by whom’ (e.g. unintentional, intentional self-harm, assault, undetermined intent, intent pending) [7]. ICD is a standardised method of coding diseases. These codes are used to bring together surveillance data. South Asia is recognised to have incomplete surveillance data, and ICD is not used everywhere [23].
Burn register	A burn register is a collection of pre-specified and systematically recorded details about burn patients [34]. Burn registers typically collect data about burn patients requiring admission to hospital for their injury. Register data can be used for surveillance.
Systematic scoping review	A systematic scoping review is a method of comprehensively drawing together literature on a research question. The research question tends to be broad [35]. It involves a number of stages (in brackets is name of the stage corresponding to the subheading in this article): developing the research question, defining which papers will be included (eligibility criteria), deciding which databases will be used to identify literature (information sources), developing the terms that will be used to search databases for literature (search strategy), screening of the search results to identify studies meeting the inclusion criteria (study records), extraction of data from included studies (data items), and drawing together the results in a meaningful way (data synthesis).
Systematic review and meta-analysis	Systematic reviews use similar methods to systematic scoping reviews. Systematic reviews tend to answer research questions that are more narrow than systematic scoping reviews [35]. They were originally developed to summarise information on medical interventions (e.g. medications) to understand if there is a benefit for patients. The process of drawing together numerical data from multiple studies is known as meta-analysis.
Preferred Reporting Items for Systematic Review and Meta-Analysis (PRISMA)	PRISMA is a set of items that should be reported for a systematic review. It was developed to promote transparent reporting of systematic reviews to ensure that they are as understandable as possible for readers. PRISMA now issues guidance for other types and aspects of reviews including protocols and systematic scoping reviews [25, 36].
Medical and social science databases	Medical and social science databases are online warehouses of published literature. They are searched during a systematic scoping review to find articles meeting the study inclusion criteria. MEDLINE is a well-known example of a database in the field of medicine.
Search strategy	A list of words based upon the research question used to search the database for articles that may meet the study inclusion criteria. The search strategy is developed in a series of steps and must be adapted for each database. Some databases use ‘index terms’ to classify their articles. Index terms may be combined with free-text words to develop the search strategy. They can be further refined with Boolean operators (e.g. AND, OR, NOT) and filters (e.g. restrictions by date or language). Systematic scoping reviews answering a medical research question tend to include their search strategy as it would be inputted into MEDLINE. This allows readers to repeat the search if they wish.

### Supplementary Information

**Additional file 1. **Completed preferred reporting items for systematic review and meta-analysis protocols (PRISMA-P) checklist.

**Additional file 2. **MEDLINE search strategy.

**Additional file 3. **Screening advice document.

## Data Availability

All data generated or analysed in this study protocol are included in this published article and its supplementary information files.

## Abbreviations

ICD: International Classification of Diseases
PRISMA: Preferred Reporting Items for Systematic Reviews and Meta-Analyses
