# Supplementary material for: Terminology and methods used to differentiate injury intent of hospital burn patients in South Asia: a systematic scoping review protocol
Source: Syst Rev. 2023 Aug 31;12:153. doi: 10.1186/s13643-023-02317-y (PMC10468849; doi:10.1186/s13643-023-02317-y)
Supplement: Supplementary file 2 — Additional file 2. MEDLINE search strategy. [file 13643_2023_2317_MOESM2_ESM.pdf]

Additional file 1. MEDLINE search strategy

Ovid MEDLINE(R) and In-Process, In-Data-Review & Other Non-Indexed Citations <1946 to XXXX>

#	Query
1	exp Burns/
2	burn*.mp. [mp=title, abstract, original title, name of substance word, subject heading word, floating sub-heading word, keyword heading word, organism supplementary concept word, protocol supplementary concept word, rare disease supplementary concept word, unique identifier, synonyms]
3	scald*.mp. [mp=title, abstract, original title, name of substance word, subject heading word, floating sub-heading word, keyword heading word, organism supplementary concept word, protocol supplementary concept word, rare disease supplementary concept word, unique identifier, synonyms]
4	thermal* injur*.mp. [mp=title, abstract, original title, name of substance word, subject heading word, floating sub-heading word, keyword heading word, organism supplementary concept word, protocol supplementary concept word, rare disease supplementary concept word, unique identifier, synonyms]
5	smoke inhalation.mp. [mp=title, abstract, original title, name of substance word, subject heading word, floating sub-heading word, keyword heading word, organism supplementary concept word, protocol supplementary concept word, rare disease supplementary concept word, unique identifier, synonyms]
6	1 or 2 or 3 or 4 or 5
7	exp Heartburn/
8	heartburn.mp.
9	heart burn.mp.
10	exp Burnout, Psychological/
11	burnout.mp.
12	burn* out.mp.
13	burnet*.mp.
14	burnish.mp.
15	7 or 8 or 9 or 10 or 11 or 12 or 13 or 14
16	6 not 15
17	exp Afghanistan/
18	exp Bangladesh/
19	exp Bhutan/
20	exp India/
21	exp Sri Lanka/
22	Indian Ocean Islands/
23	exp Nepal/
24	exp Pakistan/
25	afghan*.mp.
26	bangladesh*.mp.
27	bhutan*.mp.
28	india*.mp.
29	Sri lanka*.mp.
30	maldiv*.mp.
31	nepal*.mp.
32	pakistan*.mp.
33	17 or 18 or 19 or 20 or 21 or 22 or 23 or 24 or 25 or 26 or 27 or 28 or 29 or 30 or 31 or 32
34	comoros/ or madagascar/ or mauritius/ or reunion/ or seychelles/
35	afghan* hound.mp. [mp=title, abstract, original title, name of substance word, subject heading word, floating sub-heading word, keyword heading word, organism supplementary concept word, protocol supplementary concept word, rare disease supplementary concept word, unique identifier, synonyms]

36	india* ink.mp. [mp=title, abstract, original title, name of substance word, subject heading word, floating sub-heading word, keyword heading word, organism supplementary concept word, protocol supplementary concept word, rare disease supplementary concept word, unique identifier, synonyms]
37	Indiana*.mp. [mp=title, abstract, original title, name of substance word, subject heading word, floating sub-heading word, keyword heading word, organism supplementary concept word, protocol supplementary concept word, rare disease supplementary concept word, unique identifier, synonyms]
38	amerindian.mp. [mp=title, abstract, original title, name of substance word, subject heading word, floating sub-heading word, keyword heading word, organism supplementary concept word, protocol supplementary concept word, rare disease supplementary concept word, unique identifier, synonyms]
39	34 or 35 or 36 or 37 or 38
40	33 not 39
41	exp Burn Units/
42	Hospitalization/
43	Inpatients/
44	Patient Admission/
45	Registries/
46	burn unit*.mp.
47	admit*.mp.
48	admission.mp.
49	hospital*.mp.
50	inpatient.mp.
51	registry.mp.
52	registries.mp.
53	register.mp.
54	41 or 42 or 43 or 44 or 45 or 46 or 47 or 48 or 49 or 50 or 51 or 52 or 53
55	16 and 40 and 54
56	limit 55 to (english language and humans)
