# Supplementary material for: Terminology and methods used to differentiate injury intent of hospital burn patients in South Asia: a systematic scoping review protocol
Source: Syst Rev. 2023 Aug 31;12:153. doi: 10.1186/s13643-023-02317-y (PMC10468849; doi:10.1186/s13643-023-02317-y)
Supplement: Supplementary file 3 — Additional file 3. Screening advice document. [file 13643_2023_2317_MOESM3_ESM.docx]

Additional file 3.

Terminology and methods used to differentiate injury intent of hospital burn patients in South Asia: a systematic scoping review

Below are instructions for authors undertaking screening to help determine which articles should be included and excluded at the title and abstract screening phase, and the full text screening phase of the systematic scoping review.

**Title and abstract screening**

1. **Has the article already been screened?**

**Yes** – All duplicates should have been removed before being uploaded into Covidence. If you believe the article is a duplicate of another article please add a ‘note’ on Covidence stating that it is a duplicate and this will be checked by the lead reviewer. If the article is verified by the lead reviewer as a duplicate then please exclude the article.

**No** – Move to next criteria.

1. **Are the title and abstract written in English?**

**Yes** – Move on to next criteria.

**No** – Exclude article.

1. **Does the article study humans?**

**Yes** – Move on to next criteria.

**No** – Exclude article [Example: lab based studies on human or non-human tissue, animal studies].

1. **Is the article from a journal?**

**Yes** – Move on to next criteria. Check journal homepage to ensure a peer-review process is used.

**No** – Exclude article [Example: thesis, book].

1. **Is original data presented?**

**Yes** – Move on to next criteria [Example: quantitative studies, qualitative studies, case series, case report].

**No** – Exclude article [Example: review article, opinion piece, personal practice].

1. **Are cutaneous burns the focus of the study?**

**Yes** – Move on to next criteria [Example: burns to the skin from heat (thermal burn), chemicals (acid burn), radiation, electricity, friction].

**No** – Exclude article [Example: not related to a burn injury such as heartburn or professional burnout; focuses exclusively on non-cutaneous burns such as burns to the eye (ocular), internal burns (e.g. from ingestion of corrosive substances), or inhalational burns; multiple causes of injury discussed in which burns are not the main focus of the article (under 50% of cases)].

1. **Has the study been conducted in a South Asian country?**

**Yes** – Move on to next criteria. We have defined ‘South Asia’ to include the countries of Afghanistan, Bangladesh, Bhutan, India, Sri Lanka, Maldives, Nepal, and Pakistan in this review.

**No** – Exclude article.

1. **Does the study include hospital patients?**

**Yes** – Move on to next criteria. [Example: use of the word burn unit, hospital, secondary care, tertiary care, or inferred that data has been collected from a hospital].

**No** – Exclude article [Example: studies that do not focus exclusively on hospital patients such as autopsy studies, post-mortem studies, coroner’s studies, medicolegal death studies, forensic department studies, data collected from primary care, community studies].

1. **Was the study conducted on patients who had sustained burns during combat?**

**Yes** – Exclude article [Example: burn sustained during combat].

**No** – Move on to next criteria [Example: military hospital that also treat civilians, no discussion of whether the burns were sustained during combat].

1. **Is a term used that relates to intent of the burn or its classification?**

**Yes** – Include article [Example: intent, motive, unintentional, intentional, accidental, homicidal, suicidal, self-immolation, undetermined intent, or other term that the reviewer infers to relate to intent]. If an ambiguous term is used in the title or abstract [example: aetiology, cause, circumstances of the injury] then include the article for full text screening.

**No** – Exclude article.

**Full text screening**

1. **Has the article already been screened?**

**Yes** – All duplicates should have been removed at the title and abstract screening phase. If you believe the article is a duplicate of another article please add a ‘note’ on Covidence stating that it is a duplicate and this will be checked by the lead reviewer. If the article is verified by the lead reviewer as a duplicate then please exclude the article choosing the exclusion reason as ‘duplicate’.

**No** – Move to next criteria.

1. **Is the full text article written in English?**

**Yes** – Move on to next criteria.

**No** – Exclude the article choosing the exclusion reason as ‘Not in English’.

1. **Does the article study humans?**

**Yes** – Move on to next criteria.

**No** – Exclude the article choosing the exclusion reason as ‘non-human study’ [Example: lab based studies on human or non-human tissue, animal studies].

1. **Is the full text article peer reviewed?**

**Yes** – Move on to next criteria.

**No** – Exclude the article choosing the exclusion reason as ‘not a peer reviewed publication’ [Example: conference abstract, thesis, book, not from a peer reviewed journal].

1. **Are original data presented in the results section?**

**Yes** – Move on to next criteria [Example: quantitative studies, qualitative studies, case series, case report].

**No** – Exclude the article choosing the exclusion reason as ‘no original data presented’ [Example: review article, opinion piece, personal practice].

1. **Are cutaneous burns the focus of the study?**

**Yes** – Move on to next criteria [Example: burns to the skin from heat (thermal burn), chemicals (acid burn), radiation, electricity, friction].

**No** – Exclude the article choosing the exclusion reason as ‘cutaneous burns not studied’ [Example: not related to a burn injury such as heartburn or professional burnout; focuses exclusively on non-cutaneous burns such as burns to the eye (ocular), internal burns (e.g. from ingestion of corrosive substances), or inhalational burns; multiple causes of injury discussed in which burns are not the main focus of the article (under 50% of cases)].

1. **Has the study been conducted in a South Asian country?**

**Yes** – Move on to next criteria. We have defined ‘South Asia’ to include the countries of Afghanistan, Bangladesh, Bhutan, India, Sri Lanka, Maldives, Nepal, Pakistan in this review.

**No** – Exclude the article choosing the reason as ‘study not from a country in South Asia’.

1. **Does the study include hospital patients?**

**Yes** – Move on to next criteria. [Example: In methods section it is made explicit that data has been collected from a hospital].

**No** – Exclude the article choosing the reason as ‘study not based on hospital patients’ [Example: studies that do not focus exclusively on hospital patients such as autopsy studies, post-mortem studies, coroner’s studies, medicolegal death studies, forensic department studies data collected from primary care, community studies].

1. **Was the study conducted on patients who had sustained burns during combat?**

**Yes** – Exclude the article choosing the reason as ‘burns sustained during combat’ [Example: burn sustained during combat].

**No** – Move on to next criteria [Example: Burns not sustained during combat, burns sustained by civilians not engaged in combat].

1. **Does the methods or results section refer to the intent of the burn, its classification, or how intent has been determined?**

**Yes** – Include article [Example: use of a stem term or classifier for intent such as intent, motive, unintentional, intentional, accidental, homicidal, suicidal, self-immolation, undetermined intent, or other term that the reviewer infers to relate to intent]; method of determination of intent described.]

**No** – Exclude the article choosing the reason as ‘intent terminology or method of differentiation not discussed’.
